# Supplementary material for: Optical Genome Mapping in Routine Cytogenetic Diagnosis of Acute Leukemia
Source: Cancers (Basel). 2023 Apr 3;15(7):2131. doi: 10.3390/cancers15072131 (PMC10093111; doi:10.3390/cancers15072131)
Supplement: Supplementary file 1 [file cancers-15-02131-s001.zip › cancers-2241605_supplem_figures_rfinal.pdf]

cancers

MDPI

Supplementary Materials

Optical Genome Mapping in Routine Cytogenetic Diagnosis of Acute Leukemia

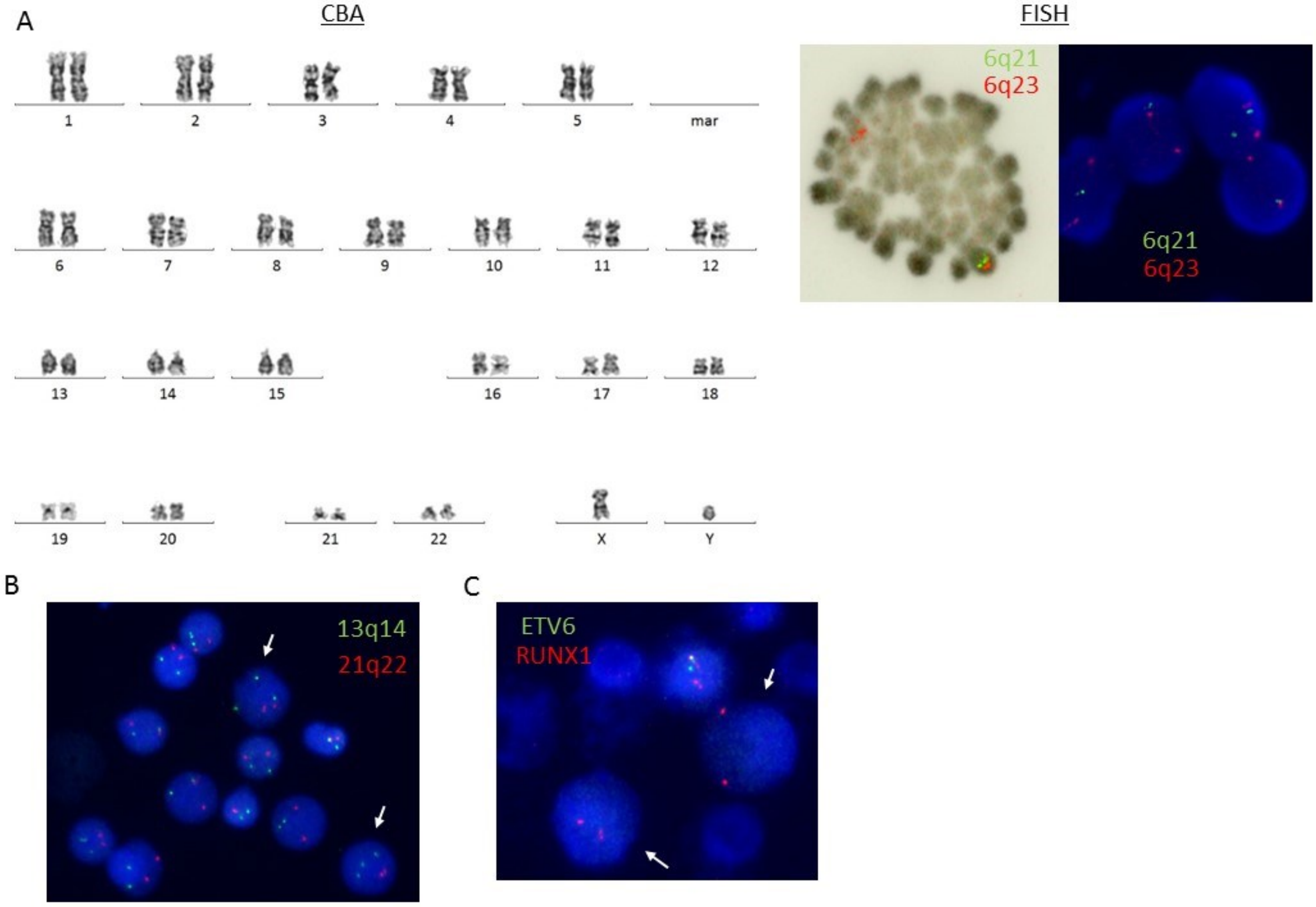

Figure S1. OGM results confirmed by FISH (×100) (A) Case #11: CBA did not detected the 40Mb deletion of the long arm of chromosome 6 revealed by OGM. FISH (XL 6q21/6q23 probe, MetaSystems) validated the deletion of the 6q21 locus on metaphases and interphase nuclei. (B) Case #10: FISH performed with XA 13/21 probe (MetaSystems) confirmed the trisomy 13. (C) Case #05: FISH performed with the LSI ETV6/RUNX1 probe (Abbott) confirmed the bi-allelic deletion of the *ETV6* locus.

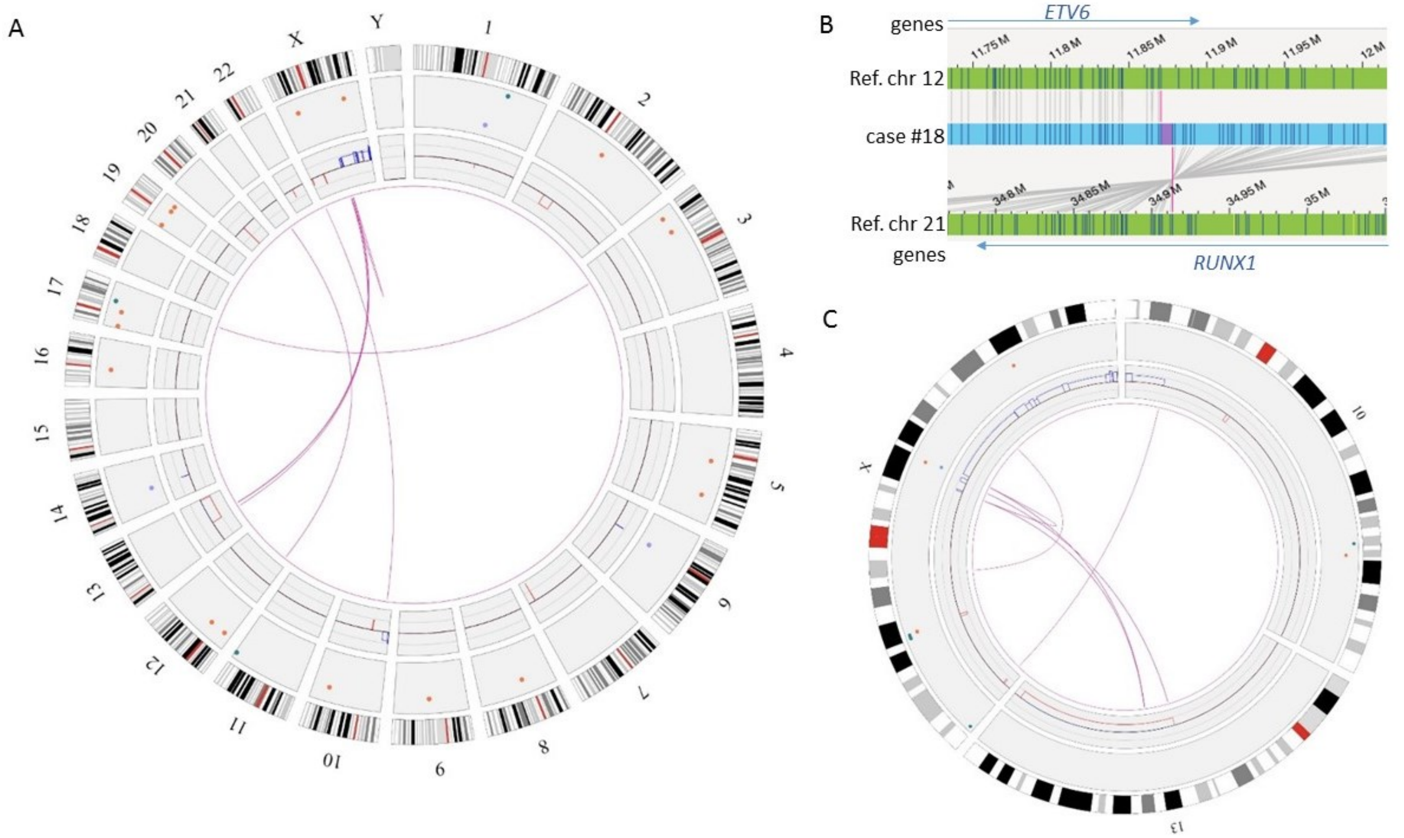

Figure S2. Translocation t(12;21) and chromotripsis. (A) Circos plot showing multiple chromosomal rearrangements in case #18. (B) Schematic representation of the alignment of a genomic map corresponding to the translocation between chromosomes 12 and 21 leading to the *ETV6::RUNX1* fusion. (C) Particular circos plot showing the chromotripsis between chromosomes X, 10 and 13.

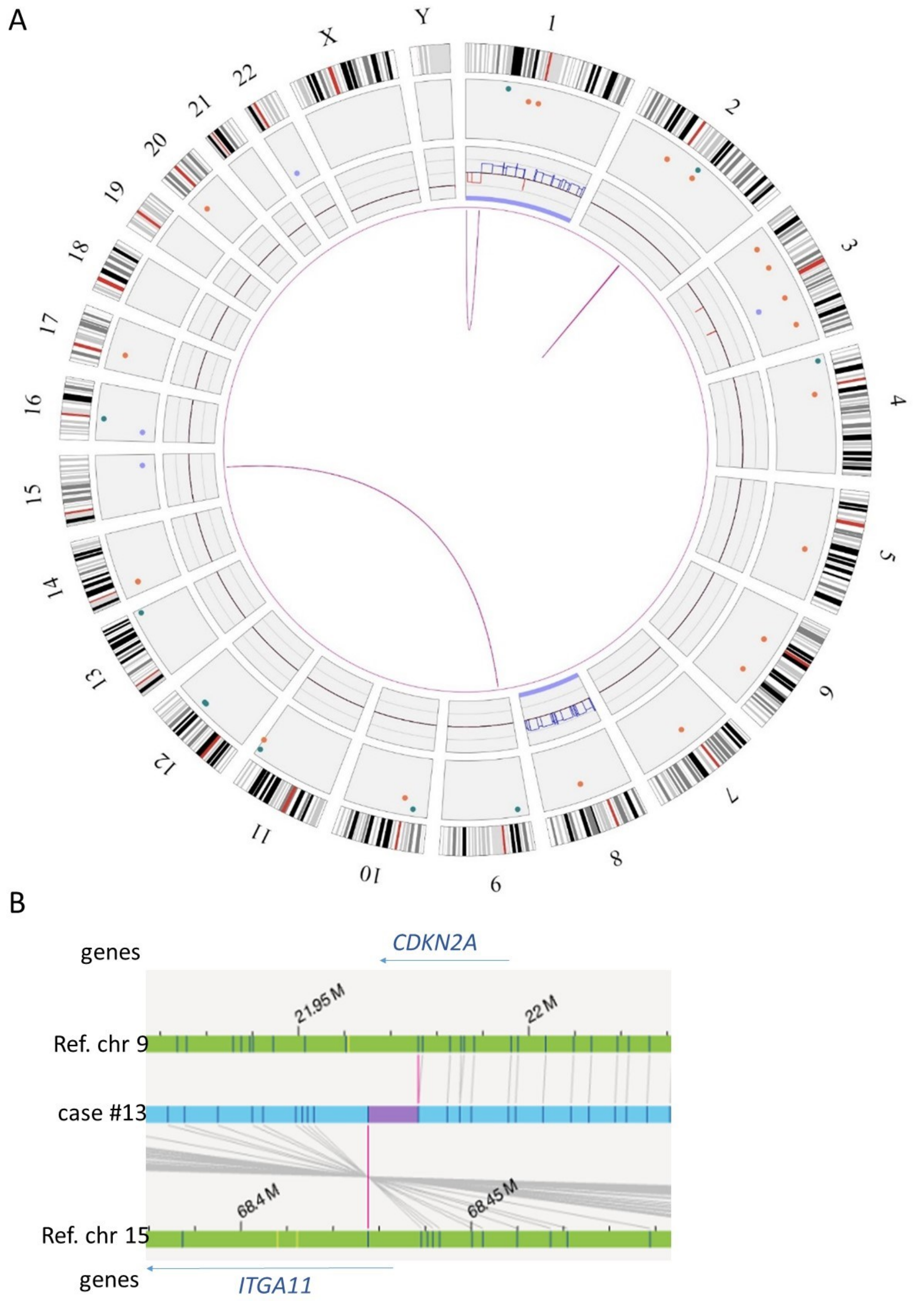

Figure S3. OGM results from case #13. (A) Circos plot showing CNV loss of the short arm of the chromosome 1 associated with a gain of the long arm, a gain of chromosome 8 and a t(9;15). (B) Schematic representation of the translocation t(9;15) showing that the breakpoint at chromosome 9 interrupts the *CDKN2A* gene.

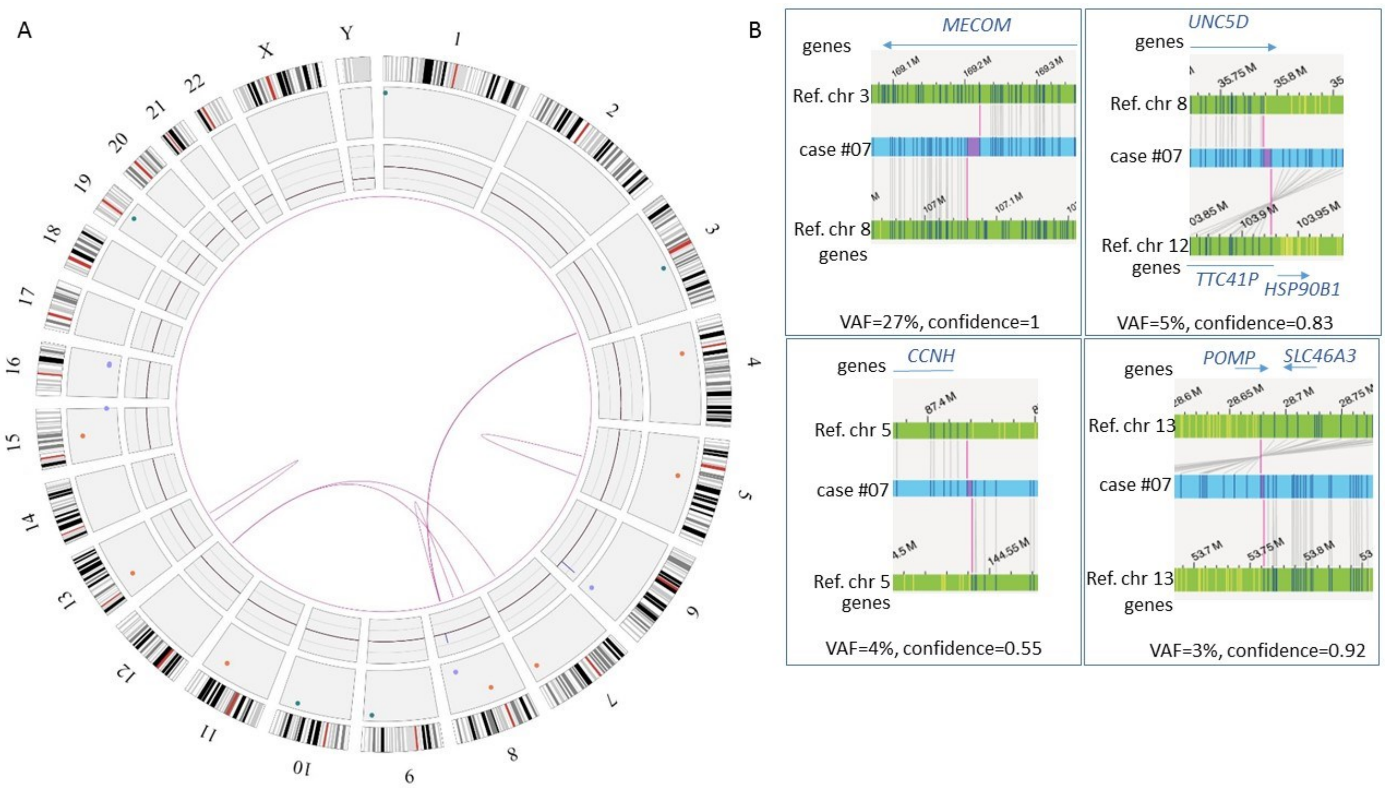

Figure S4. Example of additional abnormalities found by OGM. (A) In case #07, the circos plot showed the t(3;8), an intrachromosomal fusion in the long arm of chromosome 5, a t(7;12) (that was filtered out of the data because of confidence score = 0.26), a t(8;12) and intrachromosomal fusions in the long arm of chromosomes 8 and 13. (B) Schematic representation of the breakpoints of the t(3;8) involving the *MECOM* locus, of the breakpoints of the t(8;12) involving *UNC5D* and *TTC41P* genes, of the breakpoints of the intrachromosomal fusion in the chromosome 5 located next to the *CCNH* gene, and of the breakpoints of the intrachromosomal fusion in the chromosome 13 located next to the *POMP* gene.

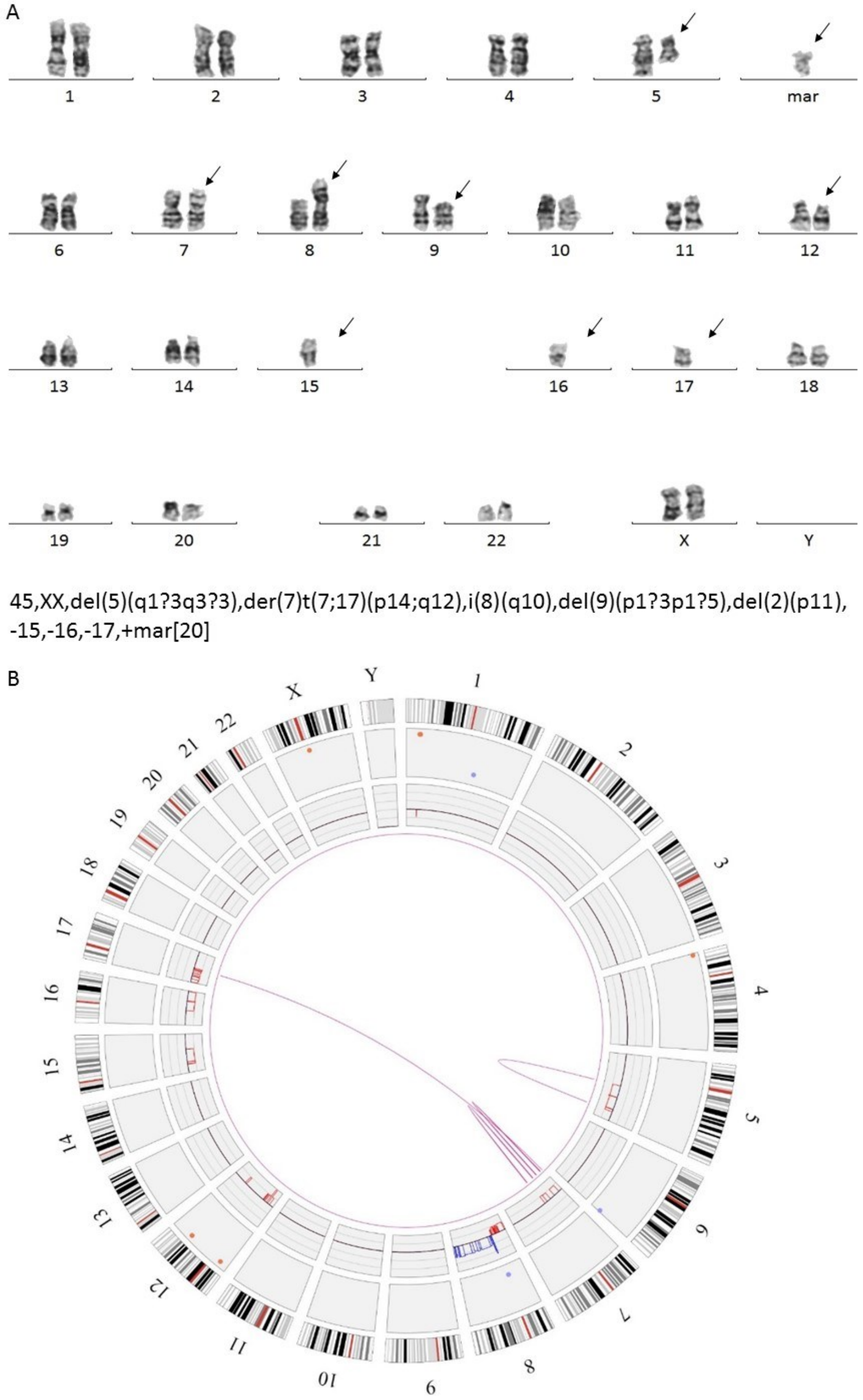

Figure S5. Example of complexity described by OGM. (A) Complex karyotype from case #02 with formula according to the ISCN (B) Circos plot from case #02 showing a CNV loss of the long arm of the chromosome 5, a CNV loss of the short arm of the chromosome 7 associated with t(7;17), a CNV gain of the long arm of chromosome 8 and several CVN losses affecting chromosomes 12, 15, 16 and 17.

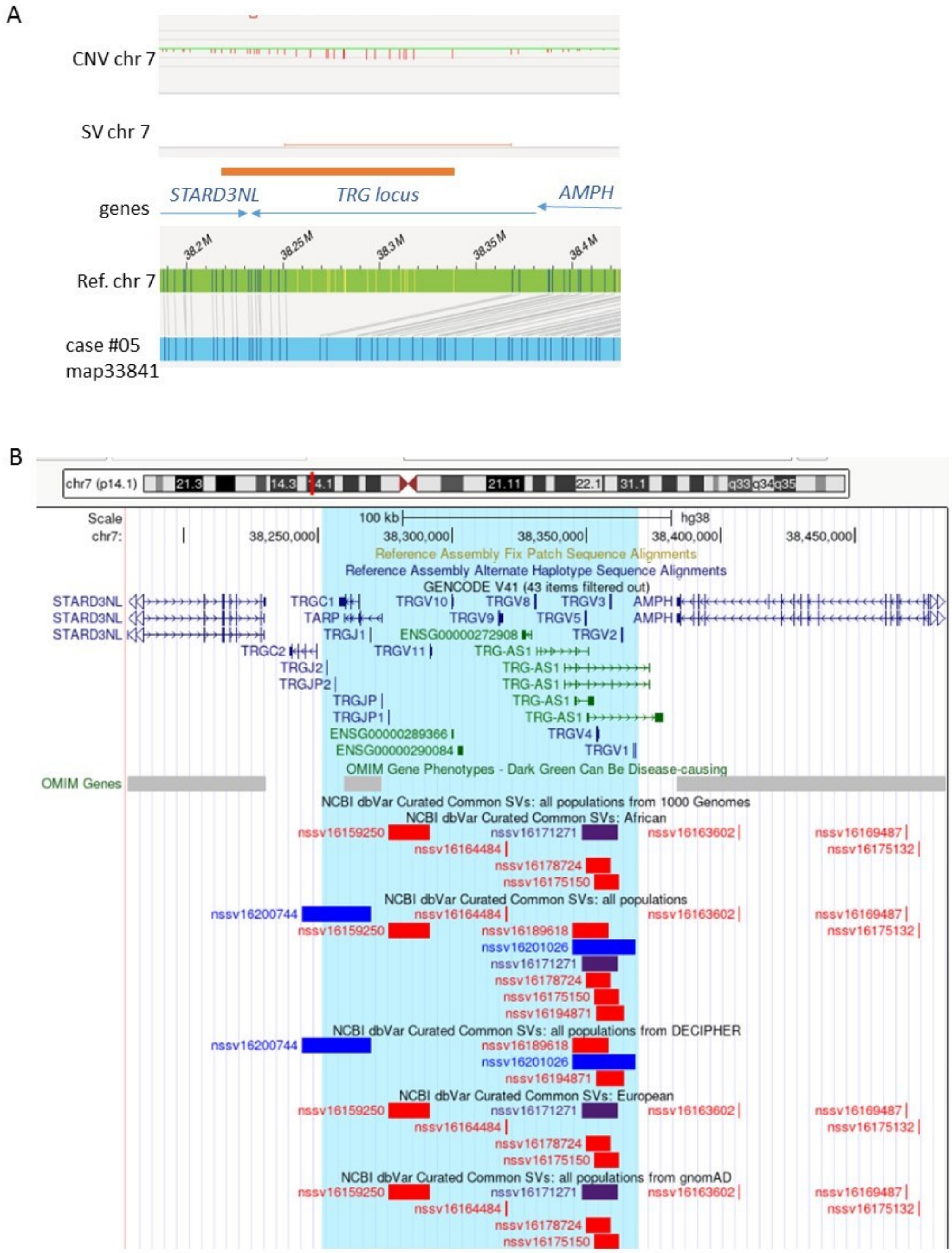

Figure S6. Deletion at the *TRG* locus(A) In case #05, map 33841 showed a deletion between positions 38.25Mb-38.37Mb on chromosome 7 overlapping the *TRG* locus, and the CNV track also showed a loss in the same region indicating that it was not a masked region (neither for SV nor CNV). After removing only the filter for the control database, the deletion was called as a SV (orange line). The software indicated that it was present in 34% of cases from the control database. (B) Visualization of the deleted region on the UCSC genome browser (http://genome.ucsc.edu) showed that the common SV in the area are smaller than the region called by the OGM analysis.

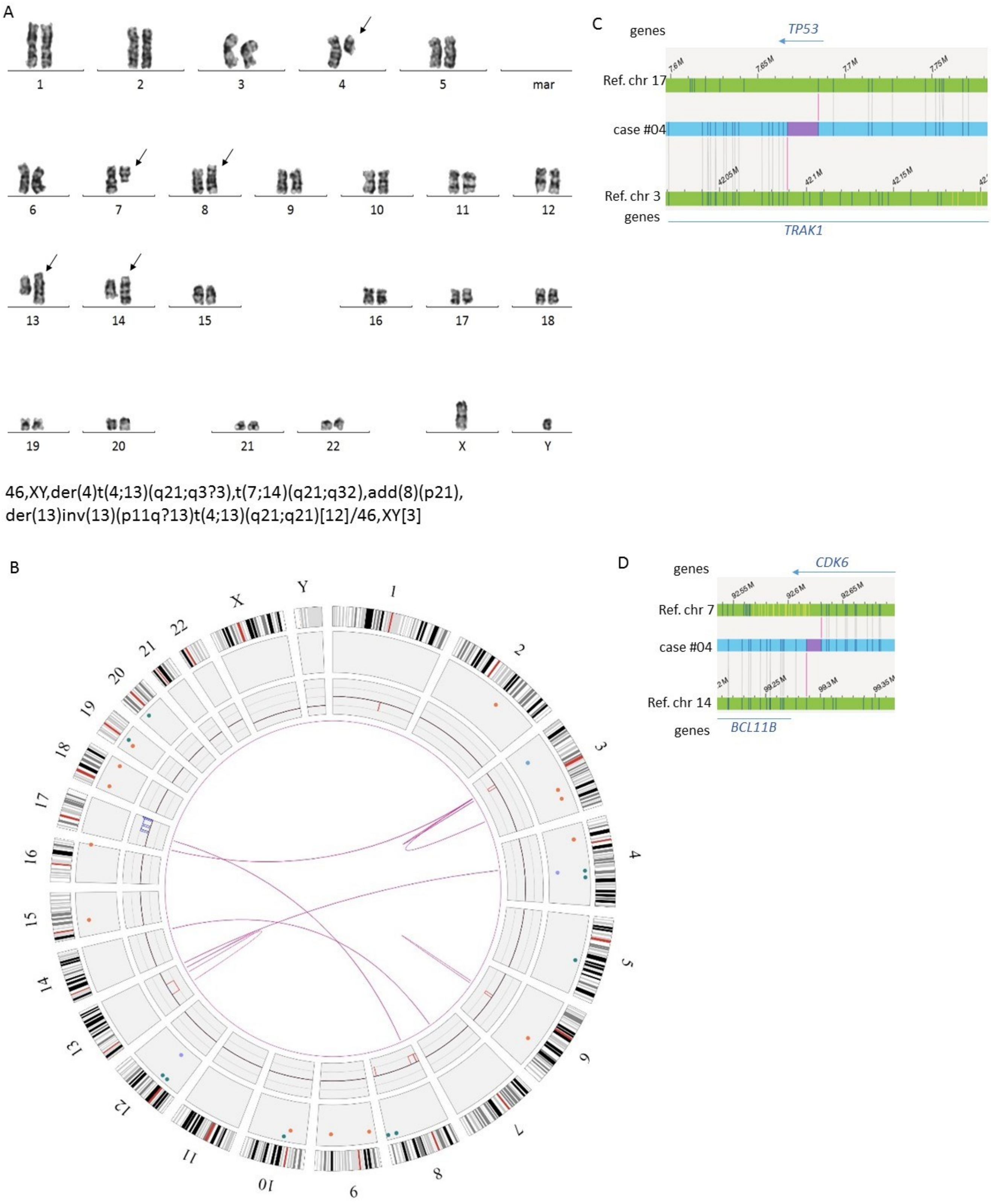

Figure S7. Translocations t(3;17) and t(7;14). (A) CBA from case #04 showing a complex karyotype with formula according to the ISCN. (B) The circos plot of OGM analysis showed 4 different translocations and several intrachromosomal SV with CNV gains or losses. (C) Schematic representation of the breakpoints of the t(3;17) involving the *TP53* and *TRAK1* genes and (D) of the t(7;14) located in the *CDK6* gene and next to the *BCL11B* gene.
